# Regulatory Elements within the Prodomain of Falcipain-2, a Cysteine Protease of the Malaria Parasite *Plasmodium falciparum*

**DOI:** 10.1371/journal.pone.0005694

**Published:** 2009-05-27

**Authors:** Kailash C. Pandey, David T. Barkan, Andrej Sali, Philip J. Rosenthal

**Affiliations:** 1 Department of Medicine, University of California San Francisco, San Francisco, California, United States of America; 2 Departments of Biopharmaceutical Sciences and Pharmaceutical Chemistry, University of California San Francisco, San Francisco, California, United States of America; 3 Graduate Group in Bioinformatics, University of California San Francisco, San Francisco, California, United States of America; 4 California Institute for Quantitative Biosciences, University of California San Francisco, San Francisco, California, United States of America; Monash University, Australia

## Abstract

Falcipain-2, a papain family cysteine protease of the malaria parasite *Plasmodium falciparum*, plays a key role in parasite hydrolysis of hemoglobin and is a potential chemotherapeutic target. As with many proteases, falcipain-2 is synthesized as a zymogen, and the prodomain inhibits activity of the mature enzyme. To investigate the mechanism of regulation of falcipain-2 by its prodomain, we expressed constructs encoding different portions of the prodomain and tested their ability to inhibit recombinant mature falcipain-2. We identified a C-terminal segment (Leu^155^–Asp^243^) of the prodomain, including two motifs (ERFNIN and GNFD) that are conserved in cathepsin L sub-family papain family proteases, as the mediator of prodomain inhibitory activity. Circular dichroism analysis showed that the prodomain including the C-terminal segment, but not constructs lacking this segment, was rich in secondary structure, suggesting that the segment plays a crucial role in protein folding. The falcipain-2 prodomain also efficiently inhibited other papain family proteases, including cathepsin K, cathepsin L, cathepsin B, and cruzain, but it did not inhibit cathepsin C or tested proteases of other classes. A structural model of pro-falcipain-2 was constructed by homology modeling based on crystallographic structures of mature falcipain-2, procathepsin K, procathepsin L, and procaricain, offering insights into the nature of the interaction between the prodomain and mature domain of falcipain-2 as well as into the broad specificity of inhibitory activity of the falcipain-2 prodomain.

## Introduction

Malaria is the most important parasitic disease in the world. *Plasmodium falciparum*, the most virulent human malaria parasite, is responsible for hundreds of millions of illnesses and about one million deaths each year (1). The control of malaria is hindered by increasing resistance to available drugs, making it important to develop new drugs to treat this disease. Among potential new targets for antimalarial chemotherapy are falcipain cysteine proteases (2). The best characterized of these proteases, falcipain-2 and falcipain-3, play key roles in the hydrolysis of hemoglobin by intraerythrocytic parasites (3–5). Inhibitors of falcipains demonstrate potent *in vitro* and *in vivo* antimalarial activity, and these proteases are the targets of efforts to develop novel cysteine protease inhibitors as new antimalarial drugs (2).

Falcipains are cathepsin L-like papain-family cysteine proteases (2). Features shared with other proteases of this sub-family include a catalytic domain of ∼30 kDa with conserved active site amino acid residues and a prodomain with potent enzyme inhibitory activity (6). We have characterized a number of unusual features of falcipains. First, folding of the mature protease is mediated by a short (14 amino acids for falcipain-2) N-terminal extension, rather than the enzyme prodomain (6, 7). Second, a 10 amino acid insertion near the C-terminus mediates interaction of the mature domain with its principal substrate, hemoglobin, and with the prodomain (8). Third, the prodomain does not have a typical signal sequence, but contains a membrane-spanning domain that predicts a type II integral membrane protein. Fourth, the falcipain prodomain is much larger than that of most other described papain-family proteases, with downstream sequence similar to papain and related enzymes, but unique upstream regions that mediate trafficking of falcipain-2 to the food vacuole, the site of hydrolysis of hemoglobin (9).

Considering its importance as a potential drug target, we were interested in evaluating the features of the falcipain-2 prodomain that mediate enzyme inhibition. We hypothesized that the inhibitory function is mediated by the downstream portion of the prodomain, which has amino acid sequence similar to that of other papain family proteases. In this region, cathepsin L-like papain family proteases, including falcipains, contain a number of conserved residues that appear to mediate interaction between the prodomain and mature protease (10), including six amino acids (ERFNIN in papain) spanning nineteen residues (11, 12) and, further downstream, four conserved amino acids (GNFD in papain) spanning seven residues (13). Conservative substitutions at these motifs are common; the sequences are ERWNIN and ANFD in cathepsin L and DRWNIN and ANLD in cathepsin K. In cathepsin L, these residues appear to stabilize the prodomain structure through the formation of salt bridges (14). To determine the roles of these conserved amino acids and other portions of the falcipain-2 prodomain in enzyme inhibition, we expressed the prodomain and a series of truncated fragments, and evaluated their inhibitory activity (15). Our results define a 61 amino acid minimum inhibitory domain, which includes the ERFNIN and GNFD motifs, that strongly inhibits falcipain-2 and many other cysteine proteases. Modeling of the falcipain-2 prodomain suggests that the prodomain covers the enzyme active site, and thereby inhibits activity by preventing substrate access.

## Results

### Identification of the Inhibitory Domain of Falcipain-2

**Figure 1:**
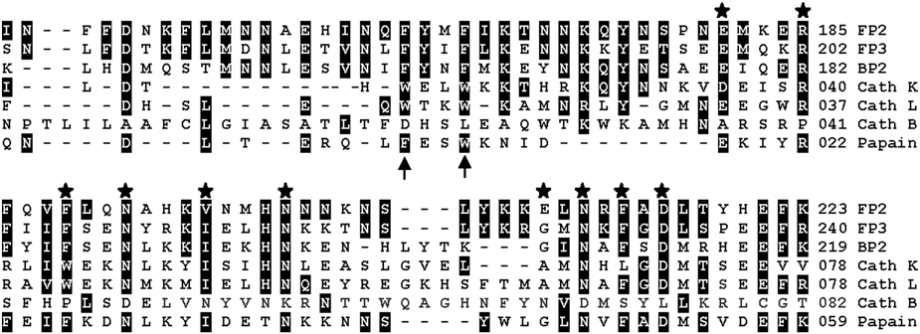
Alignment of C-terminal amino acid residues of the prodomains of falcipain-2 and related cysteine proteases. The sequences of falcipain-2 (FP2), falcipain-3 (FP3), berghepain-2 (BP2), human cathepsin K (Cath K), human cathepsin L (Cath L), human cathepsin B (Cath B), and papain were aligned using Expassy (European Bioinformatics Institute). Amino acids comprising the ERFNIN and GNFD motifs are labeled with stars, and conserved hydrophobic residues are indicated by arrows. Amino acids that are identical or similar to those of falcipain-2 are highlighted.

Falcipain-2 and homologs from related plasmodia have much larger prodomains than those of most papain-family proteases. The upstream portion of the falcipain-2 prodomain bears no obvious resemblance to sequences of non-plasmodial proteases, and mediates enzyme trafficking to the parasite food vacuole (9). In contrast, the downstream portion of the falcipain-2 prodomain is similar to that of papain, and in particular to the cathepsin L sub-family of papain-family proteases (Fig. 1). Sequence identity for this region between falcipain-2 and human cathepsin L is ∼21%, and residues that have been identified as playing key roles in the functions of papain family prodomains are generally conserved in falcipain-2 and plasmodial homologs. The well characterized ERFNIN and GNFD domains (10), which contribute to proenzyme stability, are both fully conserved in falcipain-3, but falcipain-2 differs from the consensus sequence at one ERFNIN (I→V) and one GNFD (G→E) residue. Two highly conserved Trp residues (at positions 19 and 22, of procathepsin L), which also contribute to the stability of cathepsin L sub-family proteases (12), are each replaced by Phe in both falcipain-2 and falcipain-3 (Fig. 1; falcipain-2 positions 165 and 168).

**Figure 2:**
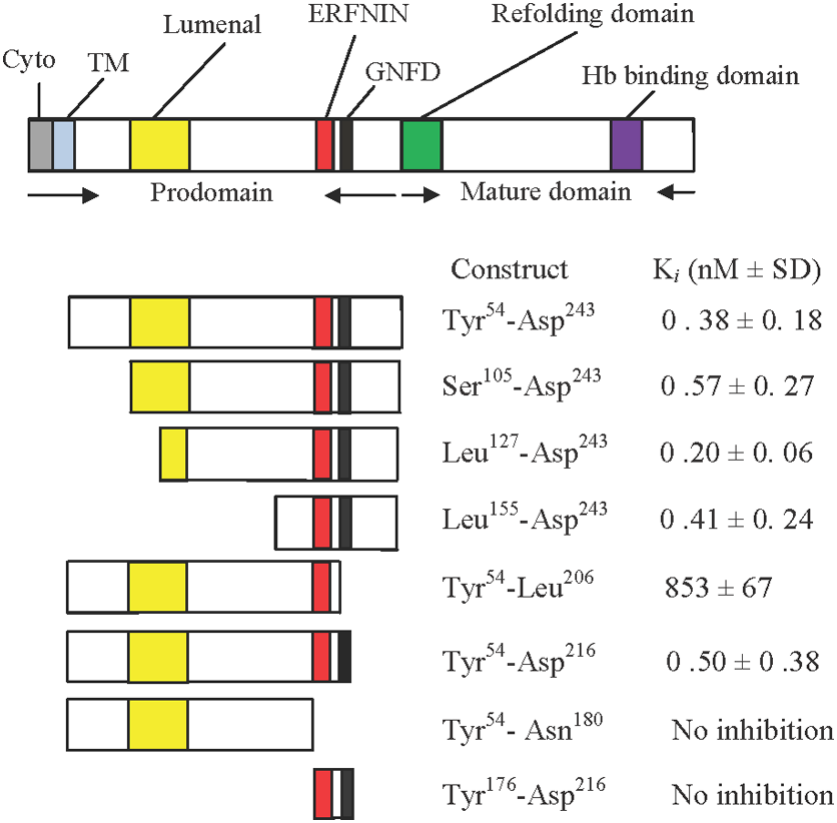
Inhibitory activity of profalcipain-2 constructs. The domains of falcipain-2 and the studied constructs are represented diagrammatically. Abbreviations: Cyto, cytosolic domain; TM, transmembrane domain; Hb, hemoglobin. The residues contained in each construct are shown, and the inhibitory capacity of mature falcipain-2 for each construct is indicated. The data provided are the Ki values for each polypeptide construct. Results are from two experiments, each performed in duplicate.

We previously showed that the prodomain of falcipain-2 is a potent reversible inhibitor of the protease (6). To characterize the requirements for inhibition, we expressed a series of prodomain fragments in *E. coli* (Figure S1) and evaluated inhibition of falcipain-2 by each of the fragments (Fig. 2). All peptides were soluble in the buffers used for our experiments and stable under our experimental conditions. As we hypothesized, the large upstream portion of the prodomain, which includes a transmembrane domain flanked by cytosolic and lumenal segments, and which mediates trafficking of falcipain-2 to the food vacuole (9), is not required for enzyme inhibition. Inhibitory potency was the same for a prodomain construct lacking only the upstream cytosolic and transmembrane domains (Tyr^54^-Asp^243^) and for constructs lacking the upstream 104 (Ser^105^-Asp^243^), 126 (Leu^127^-Asp^243^), or 154 (Leu^155^-Asp^243^) amino acids of the prodomain (Fig. 2). All of these constructs were very potent inhibitors of falcipain-2, with K*_i_* < 1 nM. Removal of the 27 C-terminal amino acids of the prodomain (Tyr^54^-Asp^216^) did not affect inhibitory potency, but removal of the 37 C-terminal amino acids (Tyr^54^-Leu^206^) led to an ∼2000-fold loss of inhibitory potency, and removal of the 63 C-terminal amino acids (Tyr^54^-Asn^180^) led to a complete loss of inhibitory activity. A peptide spanning the ERFNIN and GNFD motifs (Tyr^176^-Asp^216^) demonstrated no inhibitory activity. These results allow identification of a minimum inhibitory domain for falcipain-2 (Leu^155^-Asp^216^), which includes two hydrophobic residues (Phe^165^ and Phe^168^ in falcipain-2; Phe^182^ and Phe^185^ in falcipain-3) and the ERFNIN and GNFD motifs, all of which are highly conserved among other cathepsin L sub-family proteases. We could not directly test the inhibitory activity of this minimum inhibitory peptide, as production of the recombinant peptide was unsuccessful.

### Inhibitory Activity of the Falcipain-2 Prodomain Against Other Cysteine Proteases

**Figure 3:**
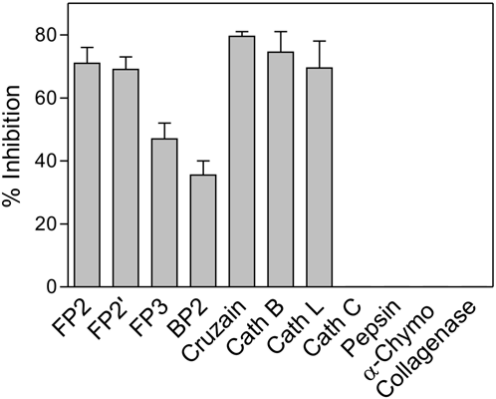
Inhibition of different proteases by the prodomain of falcipain-2. The inhibition of falcipain-2 (FP2), falcipain-2′ (FP2′), falcipain-3 (FP3), berghepain-2 (BP2), cruzain, human cathepsin B (Cath B), human cathepsin L (Cath L), bovine cathepsin C (Cath C), pepsin, α-chymotrypsin (α-Chymo), and collagenase was measured as described in Experimental Procedures. In each case activity was measured with and without the prodomain and the percentage inhibition calculated. Error bars represent standard deviations from two experiments, each performed in duplicate.

Cathepsin L sub-family protease prodomains generally inhibit only closely related proteases. For example, the prodomains of cathepsin L, cathepsin K, and cathepsin S are each potent inhibitors of all three proteases, but not of cathepsin B (10). In contrast, the falcipain-2 prodomain had a rather broad inhibitory specificity, with inhibition of the falcipain-2 homolog from *Plasmodium berghei* (berghepain-2), the *Trypanosoma cruzi* protease cruzain, cathepsin L, and cathepsin B (Fig. 3). The only tested papain-family cysteine protease that was not inhibited was the dipeptidyl peptidase cathepsin C. The aspartic protease pepsin, serine protease α-chymotrypsin, and metalloprotease collagenase were not inhibited by the falcipain-2 prodomain.

### Structural Explanation for Inhibitory Activity of Falcipain-2 Prodomain Fragments

**Figure 4:**
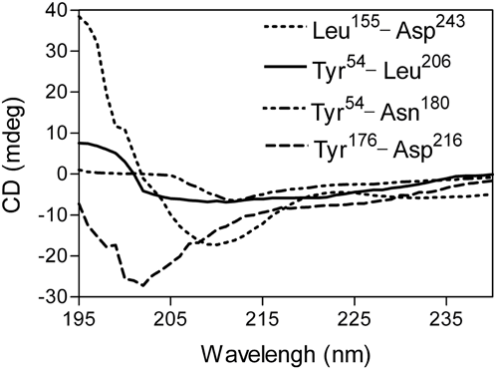
Circular dichroism analysis of prodomain constructs. Different falcipain-2 prodomain constructs (200 μg/ml) were incubated in 20 mM sodium phosphate, pH 5.8, and absorbance between 195 and 240 nm was measured.

Structure-function studies identified a discrete portion of the falcipain-2 prodomain required for inhibition of the cognate mature protease. Prior work with other cathepsin L sub-family proteases suggests key roles for conserved hydrophobic amino acids as well as the ERFNIN and GNFD motifs in maintaining prodomain structure (10). We explored the roles of different domains in maintaining prodomain structure by circular dichroism analysis (Fig. 4). Secondary structure was seen in a fragment with potent inhibitory activity (Leu^155^-Asp^243^), but not in two larger constructs that lacked any sequence downstream of the ERFNIN and GNFD motifs (Tyr^54^-Leu^206^; Tyr^54^-Asn^180^) or in a peptide spanning the ERFNIN and GNFD motifs (Tyr^176^-Asp^216^). These results indicate that the ERFNIN and GNFD motifs and an upstream region including conserved Phe residues are required for proper folding or maintenance of secondary structure of the prodomain.

### Homology Modeling of Profalcipain-2

**Figure 5:**
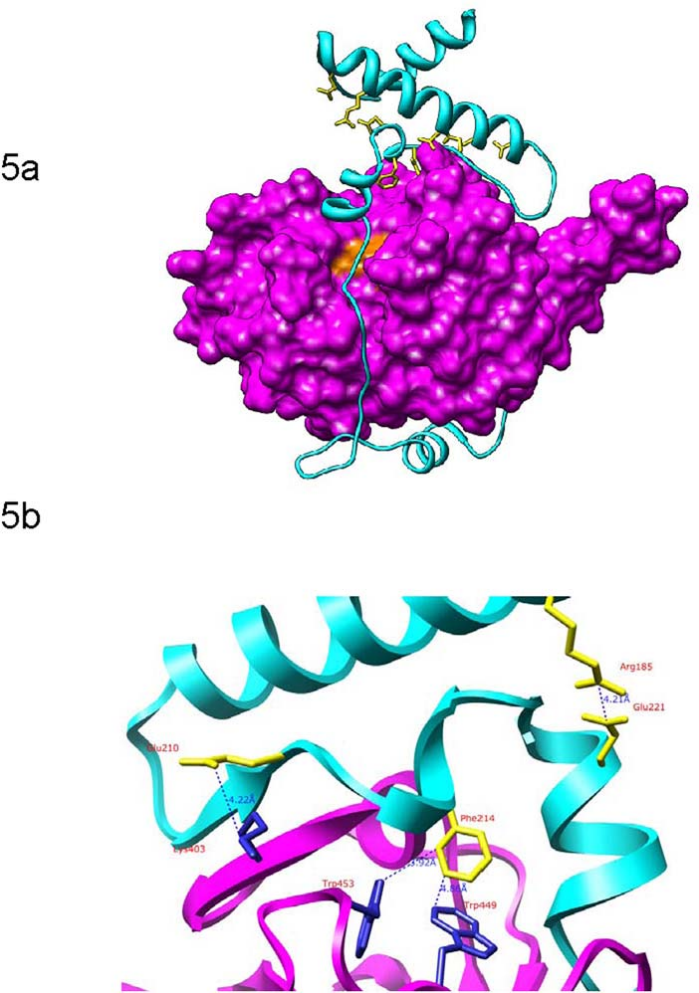
Homology model of profalcipain-2. (a) Model created using MODELLER 9v4. The 160 N-terminal residues of the prodomain are not included in the model. The prodomain (cyan) runs up the face of the mature enzyme (purple; catalytic triad residues in orange) before forming α-helices containing the conserved ERFNIN and GNFD motifs (yellow). (b) Close-up of several predicted interactions between the mature protease and the ERFNIN (R^185^) and GNFD (E^210^; F^214^) motifs. Blue dashed lines indicate presumed stabilizing interactions (both electrostatic and hydrophobic) between residues. The structure has been rotated 180 degrees around the vertical axis from its representation in figure 5a.

To explain the role of profalcipain-2 motifs in enzyme inhibition, we modeled the structure of the target falcipain-2 using the crystallographic structures of several papain-family cysteine proteases as templates. We used the software MODELLER-9v4 (16) to construct a homology model of profalcipain-2 (Fig. 5a), which aligned to mature falcipain-2, procathepsin L, procathepsin K, and procaricain at sequence identities of 100% (by definition, aligned with the sequence of the mature domain only), 30.6%, 30.9%, and 32.1% respectively (14, 20–24). The model was evaluated with DOPE (Discrete Optimized Protein Energy), a pairwise atomic distance statistical potential that assesses atomic distances in a model relative to those observed in many known protein structures (17). The DOPE Z-score of the model (−0.99) is similar to the Z-scores of all templates (cathepsin L: −1.62; mature falcipain-2: −1.13; procathepsin K −0.95; procaricain −1.25); generally, a Z-score of −1 or less indicates a relatively accurate model, with more than 80% of its Cα atoms within 3.5 Å of their correct positions (17). Additionally, a separate assessment technique, TSVMod, was applied. This method predicts the native overlap (defined as the fraction of α-carbon atoms within 3.5 Å of the native structure) of a homology model in the absence of a solved structure using support vector machine learning (18, 19). The model's predicted native overlap (0.85) was similar to that of a model of mature falcipain-2 built using the mature sections of the above templates, indicating the falcipain-2 prodomain does not contribute significantly disproportionately to the overall model error. This assessment suggests that the fold of the profalcipain-2 model is correct despite the relatively low sequence identity between the falcipain-2 prodomain and the templates.

### The Profalcipain-2 Model Suggests that the Conserved Residues Provide Stability to the Overall Fold

We examined the homology model for possible interactions involving residues in the conserved motifs. Several of these residues are highlighted in Fig. 5b. (i) The charged pair Arg^185^ and Glu^221^ appears to form a salt bridge. (ii) Glu^210^ from the GNFD motif may form a separate salt bridge with Lys^403^ in the mature domain. (iii) Phe^214^ may participate in non-polar interactions, and possibly π-bond stacking, with two tryptophan residues on the mature domain, Trp^449^ and Trp^453^. All of these interactions are also present in at least one of the templates used to build the model, although none of them is conserved across all templates.

### The Falcipain-2 Prodomain Appears to Block Substrates from Entering the Cathepsin B Active Site

**Figure 6:**
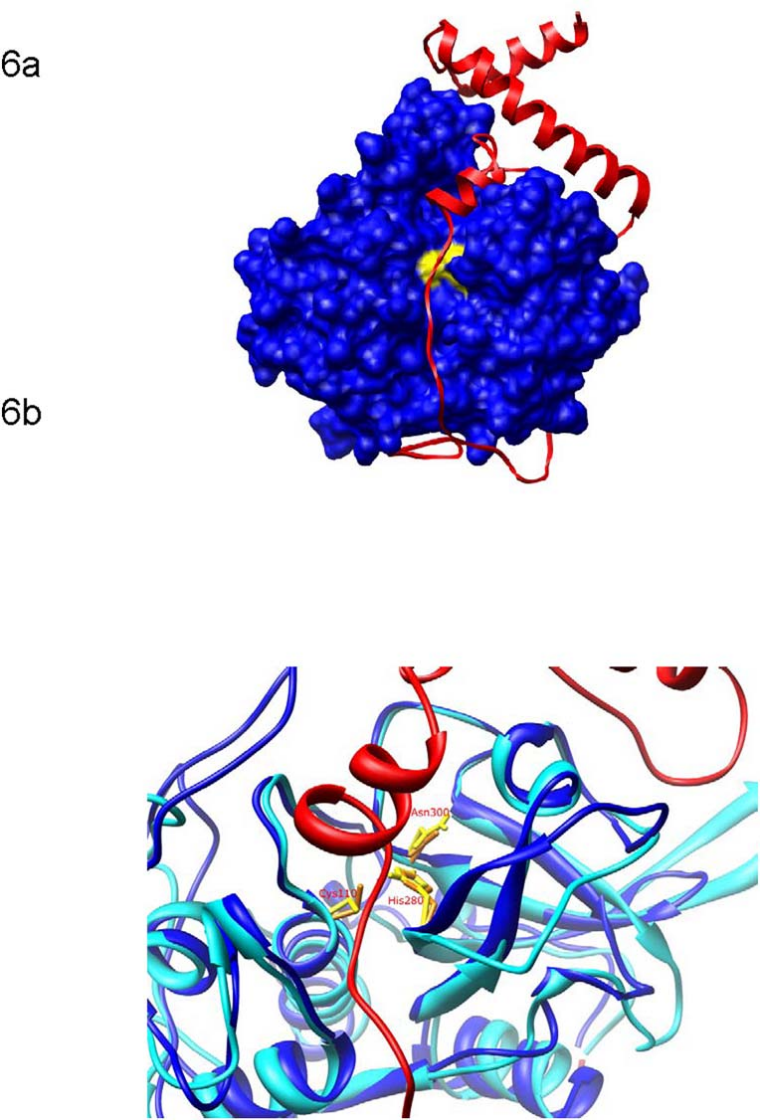
Model rationalizing the inhibition of cathepsin B by the falcipain-2 prodomain. (a) Model of the falcipain-2 prodomain (red) and mature cathepsin B (blue; catalytic triad residues in yellow). The prodomain binds to cathepsin B in a similar fashion as zymogens of other cysteine proteases, including procathepsin L and procathepsin B. (b) Structural overlay of mature cathepsin B (blue) and falcipain-2 (cyan). Catalytic triad residues are shown in the stick representation (yellow: cathepsin B; orange: falcipain-2). Cathepsin B amino acid numbering is used.

A separate homology model was constructed in which the falcipain-2 prodomain and cathepsin-B mature domain were modeled as a complex (Fig. 6a). The model was built based on an alignment of profalcipain-2 at 31.2% sequence identity with the crystallographic structure of procathepsin B (25, 26). The model received a DOPE Z score of −0.87, and a TSVMod native overlap prediction of 0.82. These scores indicate that the overall fold is correct; poor scores would have suggested that there were significant errors in the modeled structure of the prodomain, and in that case the model would not have resembled the structures of the templates on which it was based. The model suggests that the prodomain of falcipain-2 binds mature cathepsin B in a manner similar to that observed in papain family zymogens, inhibiting catalytic activity by blocking substrate access to the active site. (Fig. 6b **)**. While no structure has been solved for a propeptide in complex with an inhibited mature enzyme, it is likely that these propeptides bind to the enzymes in a conformation resembling the zymogen form (14, 25–27). This hypothesis is reflected in the model, which by construction is similar to its templates, and displays favorable stereochemistry and non-bonded atom distances as evaluated by MODELLER and DOPE.

### Differences Between the Prodomains of Falcipain-2 and Cathepsin L

**Figure 7:**
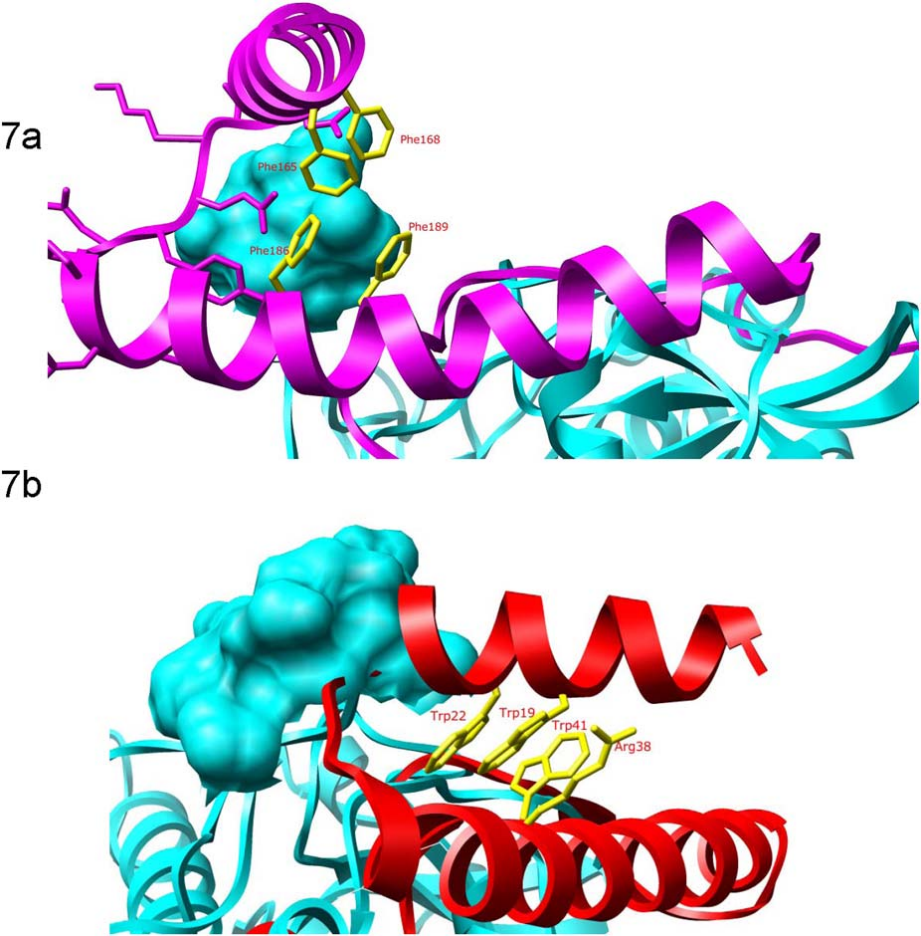
Modeled differences between falcipain-2 (a) and cathepsin L (b) prodomain binding to cathepsin B. The model predicts a helix arrangement in the falcipain-2 prodomain (purple) that prevents steric clashes with the cathepsin B occluding loop (cyan). Phe^186^ may mediate this arrangement; in cathepsin K and cathepsin L, Phe^186^ is replaced by Arg. For cathepsin L (red), there is a large steric clash between the linker joining the two cathepsin L helices and the space-filled occluding loop (cyan).

Cathepsin B activity is inhibited by the prodomain of falcipain-2 (Fig. 3) but not cathepsin L (10). To examine the structural basis of this selectivity, we compared the sequences and structures of these two proteins. Several differences were of note (Fig. 7). First, while the procathepsin L α1 helix clashes with the occluding loop region of mature cathepsin B, thus preventing binding, the equivalent helix in falcipain-2 does not. Second, Phe^186^ in profalcipain-2 participates in polar interactions with Phe^165^ and Phe^168^; in procathepsin K and procathepsin L, Phe^186^ is replaced by Arg. Third, a multiple sequence alignment reveals a conserved motif (LMNNAEHIN in falcipain-2) in the plasmodial proteases falcipain-2, falcipain-3, and berghepain-2 that represents an insertion relative to the sequences of procathepsin K and procathepsin L (Fig. 1). Finally, an apparent salt bridge (interaction not shown) is formed between Glu^210^ in the falcipain-2 prodomain and Lys^184^ in mature cathepsin B; Glu^210^ of falcipain-2 (which has replaced Gly in the GNFD motif) is replaced by Ala in cathepsin L and cathepsin K. Taken together, differences between modeled interactions for the cathepsin B mature domain with procathepsin L or profalcipain-2 appear to describe the structural basis for the observed selective inhibition of cathepsin B activity by profalcipain-2.

## Discussion

We evaluated features of the falcipain-2 prodomain that mediate enzyme inhibition. Our data show that only an ∼11 kDa C-terminal region of the prodomain is required for potent inhibition of the protease. The region includes two hydrophobic residues (both Phe in falcipain-2) and the ERFNIN and GNFD motifs, all of which are conserved among cathepsin L-like papain family proteases. The falcipain-2 prodomain also inhibited other papain family cysteine proteases, including similar cathepsin L sub-family proteases and the more distantly related cathepsin B. We explored the relevance of conserved falcipain-2 motifs by circular dichroism; the conserved residues were required to maintain the secondary structure of the prodomain. Thus, the first prerequisite for inhibitory activity was appropriate secondary structure. We also constructed a homology model of profalcipain-2 to help explain the observed experimental results. The model identified potential interactions between the inhibitory portion of the prodomain and mature falcipain-2 that appear to explain the inhibitory activity, and also the ability of the prodomain of falcipain-2, but not that of the related protease cathepsin L, to inhibit cathepsin B. Taken together, our results identify and structurally characterize a minimum inhibitory domain of the falcipain-2 prodomain, offering a starting point for new considerations for the inhibition of key proteases of malaria parasites. Indeed, small molecules that inhibit falicipains via interactions independent of the active site might offer highly specific antimalarials without detrimental effects due to inhibition of host cysteine proteases.

Results of structure-function studies were straightforward. As expected, the upstream portion of the falcipain-2 prodomain, which mediates protein trafficking (9), was not required for inhibitory activity. Indeed, only a small portion of the prodomain (Leu^155^-Asp^216^) was required for sub-nanomolar inhibition of the mature enzyme. We did not demonstrate inhibition by the isolated Leu^155^-Asp^216^ *peptide,* as production of this peptide proved difficult, but consideration of inhibition by a number of overlapping constructs (Fig. 2) clearly demonstrates that this peptide is sufficient for inhibition of falcipain-2. Circular dichroism studies suggested that the limits of the minimum inhibitory domain are dictated by requirements for appropriate folding and maintenance of a secondary structure for the inhibitory portion of the prodomain.

Due to the conserved overall fold of cathepsin precursors (10), along with the high degree of structural similarity between these proteases and mature falcipain-2 (Cα root-mean-square-deviation between falcipain-2 and cathepsin K is 0.92 Å; falcipain-2 and cathepsin L is 0.81 Å; and falcipain-2 and procaricain is 0.95 Å), profalcipain-2 is a good candidate for comparative modeling analysis. Our model has a good DOPE score, a pairwise atomic distance statistical potential that has been shown to perform well in evaluating errors in homology models (17). DOPE is particularly suited to determine the accuracy of the overall fold of a model. The DOPE score of the model of falcipain-2 was similar to those of mature falcipain-2 and procathepsin L, indicating that the overall fold of our homology model is accurate. A separate model assessment program, TSVMod, gave essentially the same results.

In our model, residues in the ERFNIN and GNFD motifs were involved in several interactions important to the stability of the falcipain-2 prodomain fold (Fig. 5b). Two interactions, Arg^185^–Glu^221^ and Phe^214^–Trp^449^/Trp^453^, appear to be conserved between falcipain-2 and cathepsin L, with equivalent residues present in procathepsin L (21). A third interaction, Glu^210^–Lys^403^, represents a unique charged pair interaction, as a Glu is found in falcipain-2, but not falcipain-3 or most related proteases, replacing the Gly in the GNFD motif. Side chain packing is the most difficult part of comparative modeling; however, in this case using the ERFNIN and GNFD motifs as well as the conserved Phe residues to guide the alignment resulted in conserved sequences across the downstream region of the prodomain (Fig. 1), increasing confidence in our predictions.

Many cathepsin L sub-family propeptides act in trans to inhibit related proteases (10). However, selectivity has been observed, and it has been demonstrated that the prodomains of cathepsin L and cathepsin K are unable to inhibit cathepsin B (25–27). Explanations for this observation include the following. First, cathepsin B lacks the ERFNIN motif, so that the protease lacks most of the α2 helix found in cathepsin L sub-family proteases. Second, cathepsin B contains a large occluding loop insertion, conferring dipeptidase activity, but preventing propeptides containing the ERFNIN motif from binding due to a steric clash between the occluding loop and the prodomain residues connecting α1 and α2 (Fig. 7b). Interestingly, selectivity for prodomain inhibition was broader for falcipain-2, as the prodomain of falcipain-2 markedly inhibited cathepsin B (Fig. 3). Our homology model adds insight to this observation.

In the model, the interaction of the helices equivalent to cathepsin L helices α1 and α2 is shifted (Fig. 7a). This shift is mediated by the presence of an additional aromatic residue in falcipain-2, Phe^186^. This residue is part of the hydrophobic core of aromatic residues that contributes to the helix interaction in cathepsin L and cathepsin K, normally mediated by two Trp residues on α1 and the Phe residue in the ERFNIN motif on α2. In falcipain-2, Phe^186^ provides additional stability, allowing α1 to shift across α2 and eliminating the steric overlap between the prodomain residues and the cathepsin B occluding loop. In procathepsin L and procathepsin K, which do not inhibit cathepsin B, Phe^186^ is replaced by Arg^38^ (procathepsin L) and Arg^41^ (procathepsin K); arginine is a basic residue that interacts less favorably with the other hydrophobic residues. (Fig. 7b).

A recent study indicated that a synthetic fifteen residue peptide (Leu^155^-Ile^169^) from a region of the falcipain-2 prodomain immediately upstream of conserved Phe residues (Phe^165^ and Phe^168^) inhibited falcipain-2 (28). The authors proposed that this segment plays an important role in inhibition of falcipain-2. However, inhibition by the peptide was at much lower (10,000 times less) potency than inhibition by our prodomain constructs, which acted at sub-nanomolar concentrations. In our model, the Leu^155^-Ile^169^ residues form the α1-helix. As noted, these residues represent an insertion relative to cathepsin L and cathepsin K. The α1 helix does not appear to actively inhibit falcipain-2, but rather appears to provide structural stability through an interaction with the α2 helix. It is thus likely that the full prodomain inhibits falcipain-2 differently from the small peptide studied recently (28), as for this peptide to come within the proximity of the falcipain-2 active site would require replacement of the α3 helix and a novel fold relative to other papain-family proteases.

Our work defines the minimum inhibitory region of the falcipain-2 prodomain. We show that several residues conserved across cathepsin L sub-family proteases are necessary for this inhibition, and present a structural model for the interaction of the falcipain-2 prodomain with both its own mature domain and that of other proteases. As natural inhibitors of parasite protease activity, propeptides present a promising basis for design of small molecules to treat malaria.

## Materials and Methods

### Reagents

Benzyloxycarbonyl-Leu-Arg-7-amino-4 methyl coumarin (Z-Leu-Arg-AMC) and Z-Phe-Arg-AMC were from Peptides International. Restriction endonucleases and polymerases were from New England Biolabs. Oligonucleotides were synthesized at the Biomolecular Resource Center, University of California, San Francisco, and by Integrated DNA Technologies. The synthetic peptide was from AnaSpec. All other reagents were from Sigma-Aldrich or as mentioned in the text.

### PCR and Sequencing

All DNA fragments were amplified from the pTOP-FP2 plasmid, which encodes the falcipain-2 gene (6). The sequence of each construct was confirmed by DNA sequencing at the Biomolecular Resource Center, University of California, San Francisco. Portions of the falcipain-2 gene were amplified using primers specific for each construct (Table S1).

### Cloning, Expression, and Refolding of Different Prodomain Constructs

Amplified DNA fragments were digested with *Bam*HI and *Hin*dIII, ligated into digested plasmids (pRSET-B; Invitrogen) and used to transform AD (DE3) pLys *E. coli* (Invitrogen). Cells were induced with β-D-thio-galactopyranoside, and recombinant proteins were solubilized in 8 M urea, 20 mM Tris-Cl, pH 8.0 at room temperature for 60 min with gentle shaking. Insoluble material was separated by centrifugation at 27,000 g for 30 min at 4°C. For the purification of the recombinant protein, the supernatant was incubated with nickel-nitrilotriacetic acid resin (Ni-NTA; Qiagen) and purified under denaturing conditions, as previously described (6). Ni-NTA purified propeptides were bound to SP-sepharose columns (Amersham Bioscience) and eluted by a step-wise gradient of 0-1 M NaCl in 8 M urea, 20 mM Tris-Cl, pH 8.0. The denatured proteins were diluted 100-fold (final concentration 20 μg/ml) in 100 mM Tris-Cl, 1 mM EDTA, 250 mM L-arginine pH 9.0, refolded at 10–12°C for 20 h, and concentrated using a 10 kDa cut-off membrane (Millipore) to 10 ml. Insoluble protein was removed using a 0.45 μm syringe filter (Millipore).

### Inhibition of Falcipain-2 by the Prodomain

Inhibitor kinetics were calculated as previously described (15). In brief, different concentrations of prodomain constructs (2–50 nM) were pre-incubated with 2 nM falcipain-2 in 100 mM sodium acetate, 5 mM DTT, pH 5.5 for 10 min at room temperature. The substrate Z-Leu-Arg-AMC (10 μM) was added, and fluorescence (excitation 355 nm; emission 460 nm) was continuously measured for 20 min at room temperature with a Labsystems Fluroskan Ascent spectrofluorometer. Enzyme concentration was determined by titration with the irreversible inhibitor morpholine urea-phenylanine-homophenylanine fluoromethyl ketone. K*i* values were determined by nonlinear regression analysis using PRISM (GraphPad Software).

### Inhibition of other Proteases by the Falcipain-2 Prodomain

Substrates were Z-Leu-Arg-AMC (10 μM) for falcipain-2, falcipain-3, and cruzain; Z-Phe-Arg-AMC (10 μM) for cathepsin L and cathepsin K**;** Z-Arg-Arg-AMC (10 μM) for cathepsin B; Pro-Arg-AMC (10 μM) for cathepsin C; and FITC-casein (8 μg/μl) for the other studied proteases. For each reaction, 1 μg of purified falcipain-2 prodomain (or, for controls, no prodomain) and 2-10 nM of each enzyme were incubated for 10 min in 350 μl of 100 mM sodium acetate, 5 mM DTT, pH 5.5 (for α-chymotrypsin and collagenase 10 mM Tris, pH 7.5), substrate was added, and substrate hydrolysis was monitored as described above or, for FITC-casein, as previously described (8).

### Circular Dichroism

Experiments were performed on a Jasco J-175 spectropolarimeter. Signals were monitored between 195 and 300 nm in 20 mM sodium phosphate, pH 5.8 at 20°C. Purified proteins were concentrated (200 μg/ml) using a 10-kDa cutoff Amicon ultraconcentrater (Millipore) and transferred to the phosphate buffer. All experiments were performed in a quartz cell of 1 cm path length (Hellma).

### Falcipain-2 Modeling

Falcipain-2 residues 161–484, encompassing the full mature domain and the C-terminal region of the prodomain, were aligned with procathepsin L, procathepsin K, and procaricain, at sequence identities of 20–25% in the prodomain region. 100 homology models were built based on the crystallographic structures of these proteins as templates (PDB codes were 1CS8, 1BY8, and 1PCI, respectively) and the crystallographic structure of mature falcipain-2 (1YVB), using the standard ‘automodel’ routine of MODELLER-9v4 (16). Models were evaluated with the Z-DOPE statistical potential (17) and the TSVMod protocol for predicting absolute model error (18). The model receiving the best Z-DOPE score was subjected to loop refinement of residues 15–20 (sequence NKQYNS), restraining the first 14 residues to a helical conformation, using the ‘loop’ routine of MODELLER-9v4 (19).

### Cathepsin-B Modeling

The prodomain of falcipain-2 was modeled in complex with the crystallographic structure of mature cathepsin B. The same homology modeling and loop modeling procedures were performed as for falcipain-2, here based on the crystallographic structures of the prodomain regions of procathepsin L, procathepsin K, and procaricain, and the solved structure of procathepsin B (PDB code 3PBH), as templates. Structural alignments of procathepsin L and cathepsin B in Fig. 7b were performed with the SALIGN command of MODELLER-9v4 (20).

## Supporting Information

Figure S1 Expression of profalcipain-2 constructs. Different constructs were expressed in E. coli, and purified as described in Experimental Procedures. For each construct, 4 μg of protein was solubilized in SDS sample buffer, electrophoresed in a 12 % SDS-PAGE gel, and stained with Coomassie blue.
(0.16 MB TIF)

Table S1 Primers (Forward, F and Reverse, R) used to amplify DNA encoding the constructs shown are listed. Restriction endonuclease cleavage sites are in bold type.
(0.04 MB DOC)
