# Supplementary figures and images for: Regulatory Elements within the Prodomain of Falcipain-2, a Cysteine Protease of the Malaria Parasite Plasmodium falciparum
Source: PLoS One. 2009 May 27;4(5):e5694. doi: 10.1371/journal.pone.0005694 (PMC2682653; doi:10.1371/journal.pone.0005694)

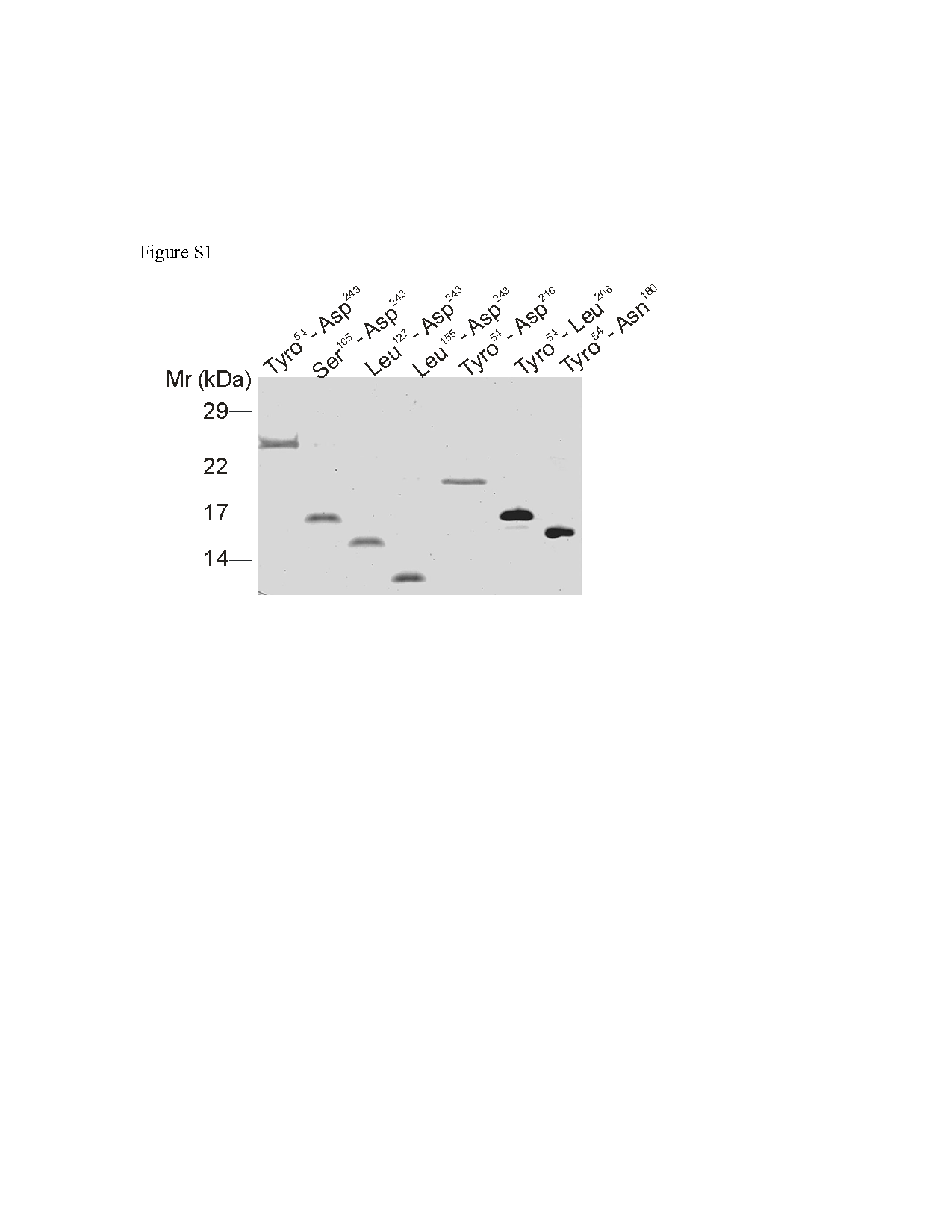

Supplement: Figure S1 — Expression of profalcipain-2 constructs. Different constructs were expressed in E. coli, and purified as described in Experimental Procedures. For each construct, 4 μg of protein was solubilized in SDS sample buffer, electrophoresed in a 12 % SDS-PAGE gel, and stained with Coomassie blue. (0.16 MB TIF) [file pone.0005694.s001.tif]
