# Supplementary material for: Regulatory Elements within the Prodomain of Falcipain-2, a Cysteine Protease of the Malaria Parasite Plasmodium falciparum
Source: PLoS One. 2009 May 27;4(5):e5694. doi: 10.1371/journal.pone.0005694 (PMC2682653; doi:10.1371/journal.pone.0005694)
Supplement: Table S1 — Primers (Forward, F and Reverse, R) used to amplify DNA encoding the constructs shown are listed. Restriction endonuclease cleavage sites are in bold type. (0.04 MB DOC) [file pone.0005694.s002.doc]

**Table S1**

| **Construct** | **Primer** |
| --- | --- |
| Tyr^54^ - Asp^243^ (F) | 5’-TTTT***GGATCC*** TATTTT TACTCCAAATTCTAGAAAAAGTG-3’ |
| Tyr^54^ - Asp^243^ (R) | 5’- TTG***AAGCTT***ATCTAATAAATATTTAGAATTCTTTAA-3’ |
| Ser^105^ - Asp^243^ (F) | 5’-GAT***GGATCC*** AGTAAAAA GAATGACATAAATAAATAC-3’ |
| Ser^105^ - Asp^243^ (R) | 5’- TTG***AAGCTT***ATCTAATAAATATTTAGAATTCTTTAA-3’ |
| Leu^127^- Asp^243^ (F) | 5’-AGGT***GGATCC***CTTAGCTTATTTAAAGAAAACACACCATC-3’ |
| Leu^127^- Asp^243^ (R) | 5’- TTG***AAGCTT***ATCTAATAAATATTTAGAATTCTTTAA-3’ |
| Leu^155^ - Asp^243^ (F) | 5’-ATTC***GGATCC***TTTAATGAATAATGCAGAACATATAAAC-3’ |
| Leu^155^ - Asp^243^ (R) | 5’- TTC***AAGCTT*** TTAATGAATAATGCAGAACATATA-3’ |
| Tyr^54^ - Leu^206^ (F) | 5’-TTTT***GGATCC***TACTCCAAATTCTAGAAAAAGTG-3’ |
| Tyr^54^ - Leu^206^ (R) | 5’-TATA***AAGCTT***TAAACTATTTTTATTATTGTTATGCAT-3’ |
| Tyr^54^ - Asp^216^ **(**F) | 5’-TTTT***GGATCC***TACTCCAAATTCTAGAAAAAGTG-3’ |
| Tyr^54^ - Asp^216^ **(**R) | 5’-TAA***AAGCTT***ATCGGCAAATCTGTTTAATTCTTTTTT-3’ |
| Tyr^54^ - Asn^180^ (F) | 5’-TTTT***GGATCC***TACTCCAAATTCTAGAAAAAGTG-3’ |
| Tyr^54^ - Asn^180^ (R) | 5’-TTTC***AAGCTT***ATTTGGAGAATTATATTGTTTATTATTAGT- 3’ |
